# Determination of Genotoxic Impurity *N*-Nitroso-*N*-methyl-4-aminobutyric Acid in Four Sartan Substances through Using Liquid Chromatography–Tandem Mass Spectrometry

**DOI:** 10.3390/molecules27217498

**Published:** 2022-11-03

**Authors:** Bin Xie, Dong Guo, Binliang Mai, Jun Fan

**Affiliations:** 1 Zhuhai Rundu Pharmaceutical Co., Ltd., Zhuhai 519041, China; 2 Department of Chemistry, School of Chemistry, South China Normal University, Guangzhou 510006, China

**Keywords:** N-nitrosamine genotoxic impurity, sartan substance, *N*-nitroso-*N*-methyl-4-aminobutyric acid, LC-MS/MS quantitative determination, multiple reactions monitoring

## Abstract

*N*-nitroso-*N*-methyl-4-aminobutyric acid (NMBA) is the third N-nitrosamine impurity found in sartans. Herein, a sensitive and stable LC-MS/MS method with multiple reactions monitoring mode has been developed for the quantitative determination of NMBA in four sartan substances. The effective separation of NMBA and sartan substances was achieved on a C18 column under gradient elution conditions. The mass spectrometry method of the atmospheric pressure chemical ionization source and internal standard method was selected as the quantitative analysis method of NMBA. Then, this proposed LC-MS/MS analysis method was validated in terms of specificity, sensitivity, linearity, accuracy, precision and stability. Good linearity with correlation coefficient over 0.99 was obtained at the NMBA concentration of 3–45 ng/mL, and the limit of quantification was 3 ng/mL. Additionally, the recoveries of NMBA in four sartan substances ranged from 89.9% to 115.7%. The intra-day and inter-day relative standard deviation values were less than 5.0%. In conclusion, this developed determination method for NMBA through liquid chromatography–tandem mass spectrometry showed the characteristics of good sensitivity, high accuracy and precision, which will be of great help for the quantitative analysis of NMBA in sartan products.

## 1. Introduction

N-nitrosamine impurities are considered as a kind of potential human carcinogens [1,2]. Carcinogenicity experiments of 4-methylnitroamino-1, 3-pydinyl butanone and N-nitroso nornicotine in animals revealed that it could induce the tumors of lung, nasal cavity and liver in F344 rats, as well as the tumors of lung, trachea and nasal cavity in Syrian gold hamsters [3]. In July 2018, the German Regulatory Agency and the European Medicines Agency discontinued sales of sartan substances in Europe, since N-nitrosodimethylamine (NDMA) was detected in sartan substances [4]. Meanwhile, the US Food and Drug Administration (FDA) began to recall the related sartan products. More kinds of N-nitrosamine impurities, such as N-nitrosodiethylamine (NDEA), N-nitrosodibutylamine (NDBA) and N-Nitrosodiisopropylamine, have been found in sartan substances [5].

**Figure 1:**
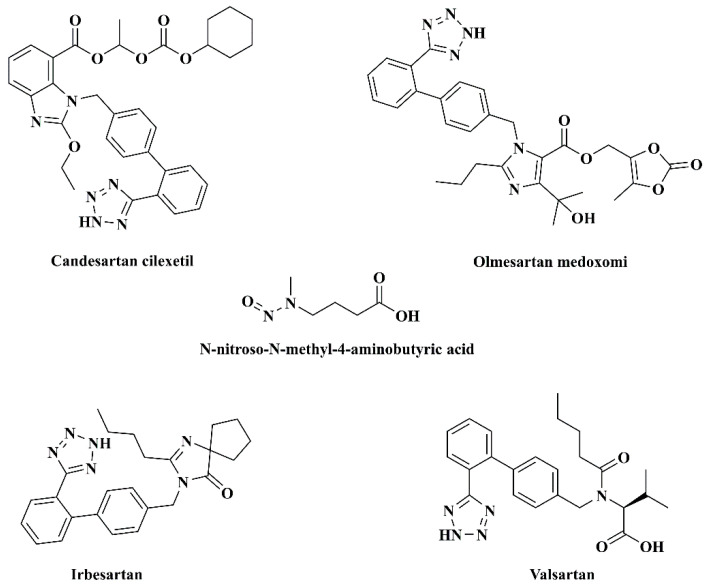
Molecular structures of NMBA and four sartan substances in this study.

*N*-nitroso-*N*-methyl-4-aminobutyric acid (NMBA, Figure 1) is the third N-nitrosamine impurity in sartan substances, and the FDA found it in the losartan potassium tablets produced by Hetero Labs Ltd. (India) in March 2019 [6]. For those containing the structure of alkyl nitrosamines, NMBA has been known to be carcinogenic in animals and potentially human carcinogens to date. NMBA, as a metabolite of several *N*-nitrosomethyl-*N*-alkylamines, could induce bladder tumors in rats after the administration in drinking water in male and female rats, thus, causing a high incidence of bladder migration cell carcinoma [7]. According to the FDA’s initial assessment results, the increased risk of cancer in patients exposed to NMBA appears to be the same for NDMA exposure, but lower for NDEA exposure. Consequently, the provisional limit values for NMBA in candesartan, olmesartan, irbesartan and valsartan are consistent with NDMA, namely, 3.0, 2.4, 0.32 and 0.3 ppm [5]. Taking into consideration the potential hazards of NMBA in safety, it is essential to establish a suitable regulatory approach for the content of NMBA in drugs.

Currently, FDA has released a new RapidFire-MS/MS method for the simultaneous detection of N-nitrosamine impurities such as NDMA, NDEA and NMBA in losartan potassium active pharmaceutical ingredients [8]. In the work by Xu et al. [9], it was reported that an LC-Q-TOF-MS method was developed to determine the content of NMBA in losartan potassium and Exforge, whereby the corresponding limit of quantitation (LOQ) was 2.1 ng/mL and the relative standard deviation (RSD) of precision was 9.8%. Additionally, an LC-MS/MS method was established for NMBA detection in losartan potassium, in which the detection time of NMBA was 2.868 min, the LOQ was 1.0 ng/mL and the RSD of peak areas for six groups of parallel samples was 4.77% [10]. Furthermore, Chidella et al. developed an LC-MS/MS quantitative analysis method for six potentially genotoxic N-nitrosamine impurities in telmisartan, in which the running time was approximately 13.5 min and a relative LOQ was 0.004 ppm [11]. However, there have been no reports of the trace analysis of NMBA in candesartan cilexetil, olmesartan medoxomil, irbesartan and valsartan through using the LC-MS/MS method to date.

Herein, an LC-MS/MS method has been developed for the determination of NMBA content in sartan substances. Subsequently, this proposed method was validated according to the International Council for Harmonization guidelines. Moreover, the NMBA contents of candesartan cilexetil, olmesartan medoxomil, irbesartan and valsartan were determined through using this proposed method.

## 2. Results and Discussion

### 2.1. Method Development

#### 2.1.1. Optimization of Chromatographic Conditions

**Figure 2:**
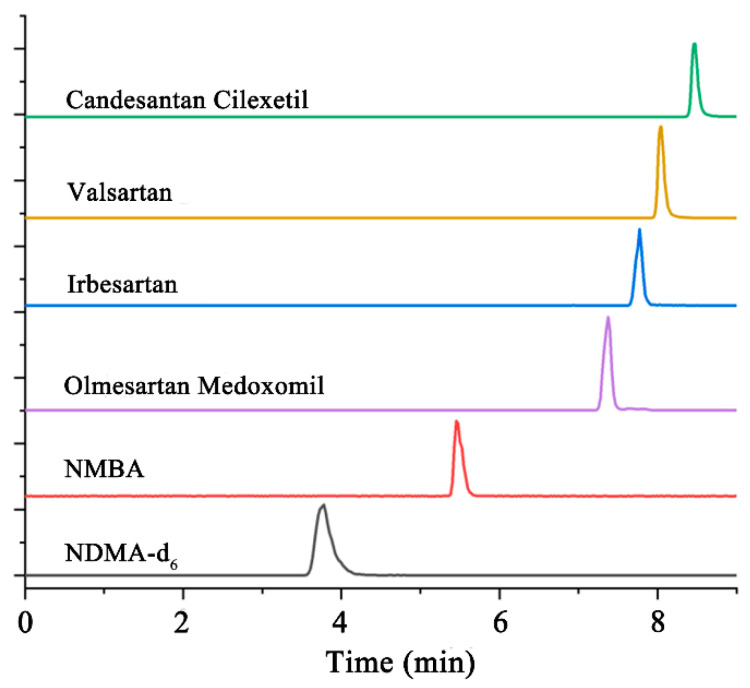
HPLC chromatograms of NDMA-d_6_, NMBA and four kinds of sartan substances on the C18 column.

In order to effectively separate NDMA-d_6_, NMBA and four kinds of sartan substances, effects of the composition and proportion of the mobile phase, as well as the content of the acid additive, were investigated on the C18 column. Water, methanol and acetonitrile were selected as the mobile phase, and the gradient elution procedure was optimized to obtain the best proportion of the mobile phase, as presented in Supporting Information Table S1. When 0.2% of formic acid was added into the mobile phase, the chromatographic peak shape of NDMA-d_6_ and NMBA were the most successful, as depicted in Figure 2. In addition, the liquid outflow mode was switched to the waste mode at 7–9 min in order to prevent the contamination of the mass spectrum with high concentrations of sartan substances.

#### 2.1.2. Optimization of Mass Spectrometric Conditions

For the sake of obtaining the best quantitative detection for NMBA, the effects of ESI and APCI sources have been discussed in detail. As presented in Table S2, the LOD and LOQ of NMBA through using the APCI source was much lower in comparison to the ESI source. This may be attributed to the small polarity of NMBA, which was difficult to be protonated under the ESI source, while a better ionization effect was found in the APCI source. Additionally, Ripollés et al. described that the APCI source had lower matrix effects for real samples by comparison to ESI [12]. Hence, the APCI source was selected for the quantitative detection of NMBA, and the corresponding parameters of the multiple reaction monitoring mode (MRM) for NMBA quantitative detection are summarized in Table 1.

#### 2.1.3. Optimization of the Quantitative Analysis Method

Herein, the comparison of the quantification of NMBA through using the external standard method and the internal standard method was conducted under the optimized chromatographic and MS detection conditions; the results are summarized in Table 2. Clearly, the correlation coefficients (*R*^2^) of NMBA through using these two methods were larger than 0.999, which indicated good linearity. In addition, the accuracy results for the 3 ng/mL spiked concentration revealed that the recoveries for the internal standard method ranged from 96.46% to 105.28%, and the recoveries for the external standard method ranged from 102.42% to 115.38%—both of which showed higher accuracy. However, the RSD values of recovery for these two methods displayed a great difference, in which the RSDs of the internal and external standard methods were 1.84%~3.96% and 5.59%~7.21%, respectively. Therefore, the internal standard method was selected for the quantification of NMBA in order to obtain good recovery and repeatability.

Taken together, the gradient elution procedure was used in the analysis of NMBA in this work. The APCI source and MRM method were used in the quantitative detection of NMBA in four sartan substances.

### 2.2. Method Validation

#### 2.2.1. Specificity

**Figure 3:**
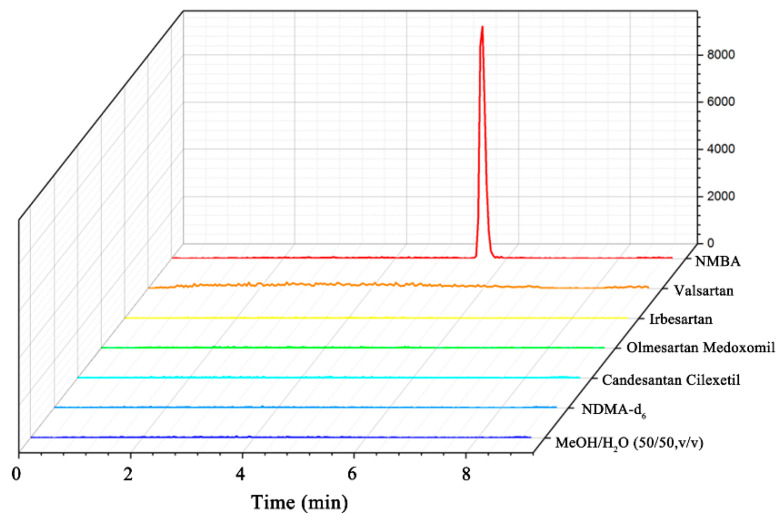
LC-MS/MS chromatograms of specificity for NMBA.

To assess the specificity of the established method, the LC-MS/MS analysis on mixtures of methanol–H_2_O (50:50, *v*/*v*), sartan matrix solutions, NDMA-d_6_ and NMBA standard solutions was performed. The criteria of specificity were determined by evaluating the interference between the NMBA standard solution, NDMA-d_6_ solution, sartan matrix solutions and blank solvent. As illustrated in Figure 3, no interference peaks were observed at the retention time of NMBA, meaning that this method had good specificity for the determination of NMBA in sartan substances.

#### 2.2.2. Linearity, LOQ and LOD

The validation results of linearity, LOQ and LOD of NMBA are presented in Figure S1 and Table 3. The concentration of NMBA was taken as the abscissa (*x*), and the ratio of the peak area of NMBA to the peak area of NDMA-d_6_ was taken as the ordinate (*y*). Standard curves of two concentration ranges, namely K1-K5 and K1-K7, were obtained in this study, and the correlation coefficients (*R*^2^) of NMBA were larger than 0.99. Additionally, the LOD and LOQ were determined by using the signal-to-noise method. Consequently, the LOD and LOQ of NMBA were 3.0 ng/mL and 0.9 ng/mL, respectively (Table 3). Notably, the low LOD and LOQ values of this LC-MS/MS method are satisfactory and sufficient for the detection of NMBA in sartan substances.

#### 2.2.3. Accuracy

The recovery and RSD of NMBA at two spiked concentrations were obtained to verify the accuracy of this method. The spiked concentrations of 3 ng/mL and 30 ng/mL were calculated through using the K1-K5 and K1-K7 standard curve, respectively. The accuracy results of NMBA in the four sartan substances are summarized in Table 4. The recoveries of NMBA at two concentration levels ranged from 89.9% to 115.7%, and the RSD values for the inter- and intra-day precision of NMBA were between 2.6% and 5.2%. The acceptance criteria for recovery were set to be in the range from 70% to 130%. Hence, this method has good accuracy and is suitable for the quantitative analysis of NMBA in sartan substances.

#### 2.2.4. Precision

The precision of this LC-MS/MS method was evaluated by determining the intra-day and inter-day precision at the 30 ng/mL spiked concentration; the precision results of NMBA in sartan substances are given in Table 5. The recoveries ranged from 95.7 to 109.2%, and the RSD values of intra-day and inter-day precision were 2.2~4.5% and 3.6~5.0%, respectively, thus, suggesting adequate precision of the method.

#### 2.2.5. Stability

The stability of NMBA and sartan substances was conducted by measuring the recoveries and RSD values of NMBA under different times, and the time was set to be 0, 2, 4, 6, 9, 12 and 24 h, respectively. As presented in Table 6, the recoveries of NMBA were between 96.0% and 105.7%, and the RSD value was calculated to be 3.6%. In conclusion, there were no significant changes for NMBA in at least 24 h.

### 2.3. Detection of Actual Samples

The proposed LC-MS/MS analytical method was used to determine NMBA in Chinese commercial sartan products, including four batches of candesartan cilexetil, three batches of olmesartan medoxomi, three batches of irbesartan and four batches of valsartan; no NMBA was detected in these drugs (Supporting Information, Table S3). Unfortunately, Chang et al. reported that 88.9% of the losartan samples contained NMBA at the content level of 27.40 ppm [13]. As reported, NMBA may be a by-product of the ring-opening reaction between N-methylpyrrolidone and nitrite during the synthesis of the sartan drug in an acidic environment [10]. Therefore, the content differences in N-methylpyrrolidone and nitrite during the production process might cause the content difference of NMBA in sartan drugs.

## 3. Experimental

### 3.1. Chemicals and Materials

Candesartan cilexetil, olmesartan medoxomi, irbesartan and valsartan were friendly provided by a local pharmaceutical company (Zhuhai, China). NMBA (purity ≥ 98.93%) and NDMA-d_6_ (purity ≥ 99.50%) were purchased from Cato Research Chemicals Inc. (Guangzhou, China). HPLC-grade acetonitrile and methanol were obtained from Merck & Co., Inc. (Whitehouse Station, NJ, USA). The Kromasil C18 column packed with octadecylsilyl stationary phase (100 × 4.6 mm, 3.5 μm) was purchased from Aone Lab Co., Ltd. (Shenzhen, China).

### 3.2. LC-MS/MS Determination of NMBA

The quantitative analysis of NMBA was conducted through using an Agilent 1260 HPLC System (Palo Alto, CA, USA) coupled with an AB SCIEX Qtrap 4000 LC-MS/MS system (Palo Alto, CA, USA). The electron spray ion (ESI) and atmospheric pressure chemical ionization (APCI) source were used as mass detectors in this study. SCIEX analysis software 1.62 was used for the system control, and MultiQuantity software 3.0 was applied in the data analysis.

### 3.3. Preparation of Standard and Sample Solutions

Standard stock solutions of NMBA and NDMA-d6 at a concentration of 0.1 mg/mL were prepared in methanol–H_2_O (50:50, *v*/*v*), respectively. Then, 30 ng/mL of the NMBA-specific solution and 50 ng/mL of the NDMA-d6 (the internal standard) solution were prepared from the stock solution, respectively. Next, a series of standard working solutions for NMBA were prepared at concentrations of 3, 6, 9, 15, 24, 30 and 45 ng/mL (named as K1-K7) with 50 ng/mL of the internal standard, respectively. In addition, 3 and 30 ng/mL of the NMBA solution under sartan substances were formulated for accuracy and precision, respectively. Furthermore, 30 ng/mL of the NMBA solution was prepared under sartan substances to assess the solution stability of the method. Additionally, these solutions were stored at −4 °C.

Moreover, 1.0 g of the sartan substance was accurately weighed and put into a 10 mL volumetric flask. Then, the NDMA-d_6_ solution (1 μg/mL, 500 μL) and 7 mL of methanol–H_2_O (50:50, *v*/*v*) were added. Next, after 30 min of sonication and 1 min of vortexing, centrifugation was conducted at 2500× *g* rpm for 10 min. Finally, the supernatant was filtered with a 0.22 μm filter membrane and transferred into vials for a chromatographic injection.

Moreover, 1.0 g of the sartan substance was accurately weighed and put into a 10 mL volumetric flask. Then, the NDMA-d_6_ solution (1 μg/mL, 500 μL) and 7 mL of methanol–H_2_O (50:50, *v*/*v*) were added. Next, after 30 min of sonication and 1 min of vortexing, centrifugation was conducted at 2500× *g* rpm for 10 min. Finally, the supernatant was filtered with a 0.22 μm filter membrane and transferred into vials for a chromatographic injection.

Moreover, 1.0 g of the sartan substance was accurately weighed and put into a 10 mL volumetric flask. Then, the NDMA-d_6_ solution (1 μg/mL, 500 μL) and 7 mL of methanol–H_2_O (50:50, *v*/*v*) were added. Next, after 30 min of sonication and 1 min of vortexing, centrifugation was conducted at 2500× *g* rpm for 10 min. Finally, the supernatant was filtered with a 0.22 μm filter membrane and transferred into vials for a chromatographic injection.

### 3.4. Method Validation

In order to evaluate and validate the performance of the established LC-MS/MS analysis method in this study, specificity, linear range, limits of quantification (LOQ) and detection (LOD), accuracy, precision and stability have been investigated in detail. LOD and LOQ were determined by considering a signal-to-noise ratio of 3 and 10, respectively. The accuracy of this method was assessed through using the recoveries of spiked samples at concentrations of 3 and 30 ng/mL. Intra- and inter-day precisions as well as the relative standard deviations (RSDs) were evaluated through sextuplicate analyses of spiked samples of 30 ng/mL over three continuous days. The stability of NMBA was evaluated by measuring the recovery and RSD value of the spiked solution over a period of time at room temperature.

## 4. Conclusions

In this work, a fast, sensitive and stable LC-MS/MS method with the MRM mode was developed for the determination of NMBA in four sartan substances. The APCI source is more suitable for the quantitative detection of NMBA in comparison to the ESI source. This method showed satisfactory selectivity and sensitivity for the determination of NMBA, and the analysis time was less than 9 min. NMBA in sartan substances at levels below the current regulatory requirement can be accurately determined through using this proposed method. As a result, this LC-MS/MS method can provide a reliable analysis method for the quality control of sartan substances and can play an important role in the potential effects on human health induced from the impurities in sartan substances.

## Figures and Tables

**Table 1:** Parameters of the multiple reaction monitoring mode.

Analyte	Precursor (m/z)	Product (m/z)	Dwell Time (ms)	Declustering Potential (V)	Collision Energy (V)
NDMA-d_6_	81.0	46.2 ^①^	200	55	25
NMBA	147.3	117.0 ^①^	200	31	10
		87.0 ^②^	200		16
Candesartan cilexetil	611.4	441.3 ^①^	200	80	16
		423.1 ^②^	200		24
Olmesartan medoxomi	559.4	541.1 ^①^	200	71	19
		207.1 ^②^	200		36
Irbesartan	429.9	207.1 ^①^	200	161	32
		402.2 ^②^	200		21
Valsartan	436.3	235.2 ^①^	200	85	24
		291.3 ^②^	200		24

^①^ Ion for quantification analyses, ^②^ qualitative ion.

**Table 2:** Recoveries and RSD values of NMBA in sartan substances through using internal and external standard methods.

Method	Linearity Range (ng/mL)	Regression Equation	*R* ^2^	Matrix	Average Recovery (%)	RSD %
Internal standard method	3~30	*y* = 0.0169*x* + 0.0018	0.9996	Candesartan cilexetil	105.3	4.0
				Olmesartan medoxomi	103.6	2.9
				Irbesartan	98.9	3.6
				Valsartan	96.5	1.8
External standard method	3~30	*y* = 2641.3*x* + 245.03	0.9998	Candesartan cilexetil	112.8	7.2
				Olmesartan medoxomi	114.6	6.9
				Irbesartan	102.4	5.6
				Valsartan	115.4	6.8

*y*: the chromatographic peak area ratio (NMBA/NDMA-d6); *x*, standard concentrations of NMBA.

**Table 3:** Calibration curves, LOD and LOQ for NMBA.

	Linearity Range (ng/mL)	Regression Equation	*R* ^2^	LOD (ng/mL)	LOQ (ng/mL)
K1-K5	3~24	*y* = 0.0178*x* + 0.0039	0.9990	3.0	0.9
K1-K7	3~45	*y* = 0.0166*x* − 0.0020	0.9994		

**Table 4:** Accuracy results of NMBA in sartan substances (mean ± SD, *n* = 6).

Matrix	Spiked Concentration (ng/mL)	Average Detected Concentration (ng/mL)	Average Recovery (%)	RSD (%)
Candesartan cilexetil	3.0035	3.2643	108.7	3.7
	30.0350	32.9359	109.7	2.6
Olmesartan medoxomi	3.0035	2.8315	94.3	3.0
	30.0350	30.3470	101.0	3.8
Irbesartan	3.0035	2.6994	89.9	3.8
	30.0350	30.6522	102.1	4.9
Valsartan	3.0035	3.4747	115.7	3.8
	30.0350	30.3651	101.1	5.2

**Table 5:** Intra-day and inter-day precision of NMBA in sartan drugs.

Matrix	Spiked Concentration (ng/mL)	Intra-Day (*n* = 6)						Inter-Day (*n* = 18)	
		Day 1		Day 2		Day 3		Average Recovery (%)	RSD (%)
		Average Recovery (%)	RSD (%)	Average Recovery (%)	RSD (%)	Average Recovery (%)	RSD (%)		
Candesartan cilexetil	30.0350	102.3	2.3	105.7	2.2	98.5	3.8	102.2	4.0
Olmesartan medoxomi		104.5	2.9	109.2	3.5	103.9	2.3	105.8	3.6
Irbesartan		95.7	3.9	97.5	4.5	102.5	3.8	98.6	5.0
Valsartan		101.3	3.4	102.6	3.4	107.0	4.5	103.6	4.3

**Table 6:** Stability results of the proposed method.

Time (h)	Spiked Concentration (ng/mL)	Average Detected Concentration (ng/mL)	Average Recovery (%)	RSD (%)
0	30.0350	29.8781	99.5	3.6
2		29.4553	98.1	
4		29.2079	97.3	
6		28.8273	96.0	
9		31.7491	105.7	
12		31.2361	104.0	
24		30.3306	101.0	

## Data Availability

In this work, Data is contained within the article or supplementary material.

## Supplementary Materials

The following supporting information can be downloaded at: https://www.mdpi.com/article/10.3390/molecules27217498/s1, Table S1: HPLC gradient in this work; Table S2: Comparison of LOD and LOQ for NMBA by APCI and ESI source; Table S3: Determination results of NMBA content of commercial sartans substances; Figure S1: Standard curves of NMBA.

## Abbreviations

**APCI**	Atmospheric pressure chemical ionization
**ESI**	Electron spray ion
**LC-MS/MS**	Liquid Chromatography–Tandem Mass Spectrometry
**LOD**	Limit of detection
**LOQ**	Limit of quantitation
**NDBA**	N-nitrosodibutylamine
**NDEA**	N-nitrosodiethylamine
**NDMA**	N-nitrosodimethylamine
**NMBA**	*N*-nitroso-*N*-methyl-4-aminobutyric acid
**RSD**	relative standard deviation
