# Supplementary material for: Determination of Genotoxic Impurity N-Nitroso-N-methyl-4-aminobutyric Acid in Four Sartan Substances through Using Liquid Chromatography–Tandem Mass Spectrometry
Source: Molecules. 2022 Nov 3;27(21):7498. doi: 10.3390/molecules27217498 (PMC9654383; doi:10.3390/molecules27217498)
Supplement: Supplementary file 1 [file molecules-27-07498-s001.zip › molecules-1999133-supplementary.pdf]

Supplementary Material

Determination of Genotoxic Impurity *N*-nitroso-*N*-methyl-4-aminobutyric Acid in Four Sartan Substances through Using Liquid Chromatography–Tandem Mass Spectrometry

Bin Xie [1], Dong Guo [2], Binliang Mai [2] and Jun Fan [2,*]

[1] Zhuhai Rundu Pharmaceutical Co. Ltd., Zhuhai 519041, China
[2] Department of Chemistry, School of Chemistry, South China Normal University, Guangzhou 510006, China
* Correspondence: fanj@scnu.edu.cn; Tel./Fax: +86-20-39310187

Table S1. HPLC gradient in this work.

t (min)	Mobile Phase A* (%)	Mobile Phase B *(%)
0.0	95	5
1.0	95	5
5.0	0	100
6.5	0	100
6.6	95	5
9.0	95	5

*Mobile phase A, formic acid/water =2/998 (*v*/*v*);

*Mobile phase B, acetonitrile/methanol =200/800 (*v*/*v*).

Table S2. Comparison of LOD and LOQ for NMBA by APCI and ESI source.

Ion Source	NMBA		
	Response*	LOD (ng/mL)	LOQ (ng/mL)
$APCI^+$	85327	0.9	3.0
ESI^+	69654	2.0	5.0

*Response at 30 ng/mL NMBA.

Table S3. Determination results of NMBA content of commercial sartans substances.

Sample	Batch Number	Detect Concentration (ng/mL)
Candesartan cilexetil	61118110102	N.D.
	61118110103	N.D.
	61118110104	N.D.
	61118110105	N.D.
Olmesartan medoxomi	80319010514	N.D.
	80319020501	N.D.
	80319020502	N.D.
Irbesartan	80319010514	N.D.
	80319020501	N.D.
	80319020502	N.D.
Valsartan	67819030610	N.D.
	67819030611	N.D.
	67819030612	N.D.
	67819030613	N.D.

N.D.—not detected.

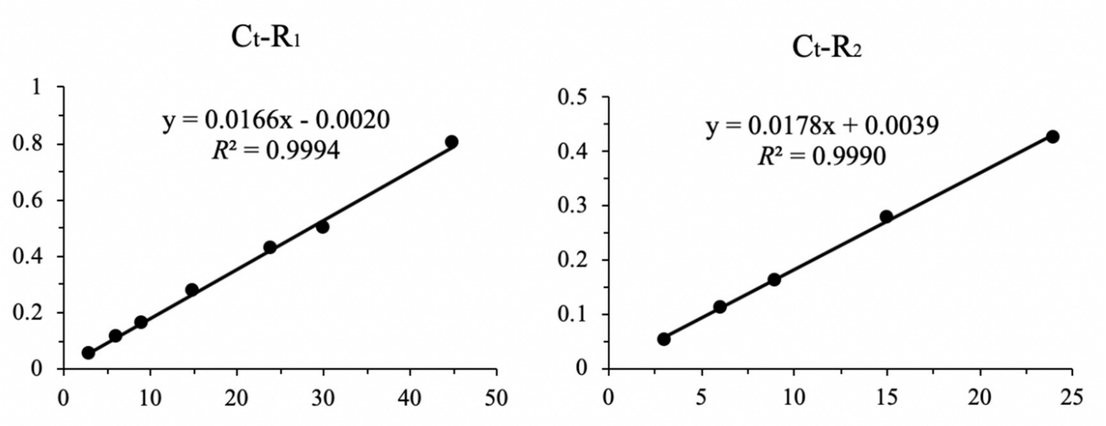

Figure S1. Standard curves of NMBA. (**A**) K1–K7; (**B**) K1–K5.
